# Transcriptome Analysis Reveals a Major Gene Expression Pattern and Important Metabolic Pathways in the Control of Heterosis in Chinese Cabbage

**DOI:** 10.3390/plants12051195

**Published:** 2023-03-06

**Authors:** Ru Li, Shanshan Nie, Ning Zhang, Min Tian, Lugang Zhang

**Affiliations:** State Key Laboratory of Crop Stress Biology for Arid Area, College of Horticulture, Northwest A&F University, Xianyang 712100, China

**Keywords:** Chinese cabbage, heterosis, transcriptome analysis, differentially expressed genes, metabolic pathway

## Abstract

Although heterosis is commonly used in Chinese cabbage, its molecular basis is poorly understood. In this study, 16Chinese cabbage hybrids were utilized as test subjects to explore the potential molecular mechanism of heterosis. RNA sequencing revealed 5815–10,252 differentially expressed genes (DEGs) (female parent vs. male parent), 1796–5990 DEGs (female parent-vs-hybrid), and 2244–7063 DEGs (male parent vs. hybrid) in 16 cross combinations at the middle stage of heading. Among of them, 72.83–84.20% DEGs conformed to the dominant expression pattern, which is the predominant expression pattern in hybrids. There were 13 pathways in which DEGs were significantly enriched in most cross combinations. Among them, the plant–pathogen interaction (ko04626) and circadian rhythm-plant (ko04712)were significantly enriched by DEGs in strong heterosis hybrids. WGCNA also proved that the two pathways were significantly related to heterosis in Chinese cabbage.

## 1. Introduction

Heterosis is a phenomenon that the hybrid is superior to their parents in biomass, growth rate, yield, stress resistance, fecundity, quality, environmental adaption [1,2,3,4]. In historical process, heterosis phenomenon was perceived in various terms in different civilizations. By the 1870s, Darwin fully described the term “heterosis”, systematically observed the growth patterns of more than 60 plants, and concluded that “Hybridization is often beneficial to plants, and selfing is bad for plants” [5,6]. For many years since its discovery, heterosis was widely used as a breeding method to improve the yield and quality in many crops. At present, the utilization of heterosis is one of the most successful biological phenomena used by human beings in agricultural production [7].

Subsequently, numerous academics have investigated heterosis and advanced many hypotheses. Among them, the dominance hypothesis, overdominance hypothesis and epistasis hypothesis were widely accepted and served as the foundation for heterosis research [8,9,10,11,12,13]. The heterosis of plants was not adequately and rationally explained by any of these hypotheses or perspectives, regardless of how divergent they were. SSR markers and quantitative trait loci (QTL) mapping have increasingly become a standard tool for examining the genetic basis in hybrids due to the development of PCR [14]. Yu et al. summarized the QTL effect on heterosis based on 35 studies and found that dominance and epistasis had equal proportions in these studies, suggesting that the results of QTL mapping differed among species and even within different groups of the same specie [15]. Therefore, SSR markers and QTL mapping are insufficient to comprehensively explain the heterosis.

RNA sequencing has become a popular tool for studying heterosis due to the development of science and technology. Currently, RNA sequencing technology is being utilized to investigate the causes of heterosis in rice [16,17], rape [18,19], Arabidopsis [20,21], maize [22,23], wheat [24,25] and cotton [26]. On the one hand, special gene expression patterns based on gene expression level have been investigated in parents and hybrids [1,27]. It was found that the proportion of allelic additively expressed genes is positively associated with hybrid yield and heterosis in maize [22]. The coexistence of nonadditive DEGs including high-parent dominance, low-parent dominance, overdominance and underdominance, was observed in the F_1_ hybrid from *Brassica napus* and *B. rapa*, which were potentially related to heterosis [19]. On the other hand, enrichment analysis of differentially expressed genes between hybrids and their parents revealed special metabolic pathways connected to heterosis [28,29]. In Arabidopsis, integrating circadian rhythm and light signaling into ethylene production is a regulatory module of complex biological networks, leading to biomass heterosis [29]. RNA sequencing offers a new method for investigating the molecular mechanism of heterosis, selecting parent and predicting heterosis [30,31].

Chinese cabbage (*Brassica rapa* L. ssp. *pekinensis*), which belongs to the Brassica species in Cruciferae family, is one of the most widely cultivated vegetable crops in Asia [32]. In the breeding process, heterosis is an important selection criterion in Chinese cabbage. Yueet al. identified four heterotic quantitative trait loci (QTL) that could explain a part of the phenotypic variation using QTL-seq and Graded Pool-seq [33]. Li et al. identified differentially expressed microRNAs by small RNA sequencing and miRNA target genes by degradome sequencing in a F_1_ hybrid [34]. These results explain heterosis from different aspects, but only one or a few special hybrids were used. It is possible that these results only exist in these materials and are not universal. In this study, 16 hybrids hybridized by four female parents and four male parents were utilized as materials to investigate the molecular mechanism of heterosis in Chinese cabbage.

## 2. Results

### 2.1. Overview of RNA-seq Analysis

**Figure 1:**
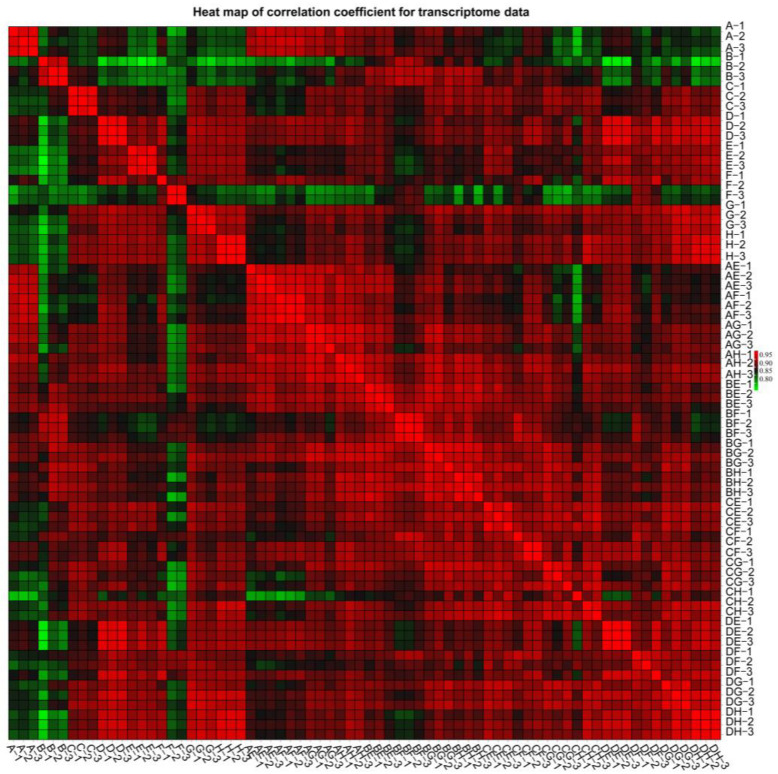
Heat map of correlation analysis based on transcriptome data in all transcripts.

The leaf samples in hybrids and parents were harvested and used for RNA-seq analysis. In this stud, high-quality RNA was extracted, and 72cDNA libraries were separately prepared. After preliminary filtration, clean reads ranged from 6.00 billion to 16.25 billion bp (Table S1). HQ clean reads ranged from 5.84 billion to 15.78 billion bp were obtained by further strict filtration. Among them, 75.62–80.01%were mapped to the available *Brassica rapa* genome V3.0 using HISAT2 (Table S2). Then, a total of 26,266–32,338 genes were assembled for hybrids and parents (Table S3). All the correlation coefficients between different biological replicates showed higher than 0.90; thus, transcriptome data and each cDNA sample had high replicability (Figure 1).

### 2.2. Analysis of Differentially Expressed Genes in Hybrids and Parents of Chinese Cabbage

Through filtering by the criteria that FDR ≤ 0.05, the differentially expressed genes (DEGs) between different groups were identified. In 16 cross combinations, there were 5815–10,252 DEGs (female parent vs. male parent), 1796–5990 DEGs (female parent vs. hybrid), and 2244–7063 DEGs (male parent vs. hybrid) (Table 1). In every cross combination, the number of DEGs between parents was higher than that between parents and hybrid. There were many genes that differed between parents and hybrid among the DEGs between parents, accounting for 51.93–76.79% of the total. Therefore, DEGs between hybrid and their parents are mostly composed of genes that are also differentially expressed between parents.

### 2.3. Expression PatternAnalysis of Differentially Expressed Genes

**Figure 2:**
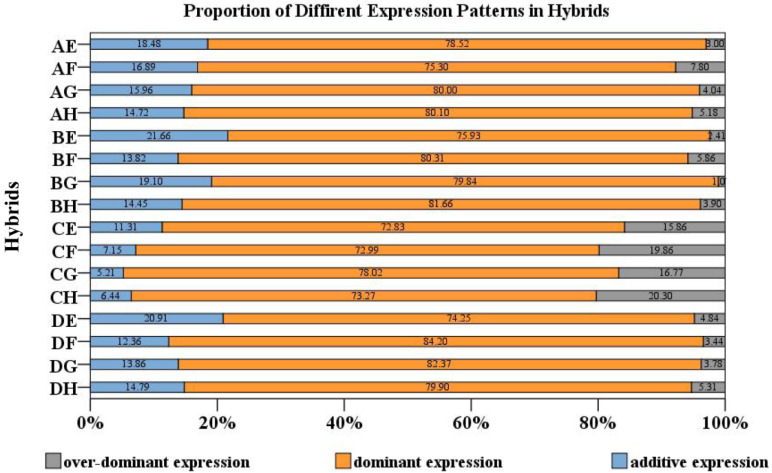
The percentages (%) in the graph represent the ratios ofthe additive expression pattern, dominant expression pattern and over-dominant expression pattern.

For further analysis of DEGs, the genes were divided into 12 expression patterns based on the expression level of the parents and hybrid (Table 2). Genes in P1 and P2 showed an additive expression. Genes conformed to P3–P6showeda dominant expression. The over-dominant expression was observed in P7–P12. Genes with dominant expression patterns accounted for 72.83–84.20% of the total in Chinese cabbage, indicating that the dominance impact is important in heterosis (Figure 2). Among them, there were, on average, 1143 genes that conformed to P3, 1391 genes that conformed to P4, 669 genes that conformed to P5, and 1184 genes that conformed to P6 in cross combinations (Table 2). The genes that exhibited an additive expression pattern accounted for only 5.21–21.66% of the DEGs. The genes that exhibited an over-dominant expression pattern accounted for 1.07–20.30% of the DEGs (Figure 2).

### 2.4. MetabolicPathways Involved in Heterosis of Chinese Cabbage

DEGs between parent and hybrid were subjected to enrichment analysis to investigate the GO terms connected to heterosis. DEGs in 16 cross combinations were classified into 46–50 functional groups including 21 GO terms in the biological process group, 17 GO terms in the cellular component group and 12 GO terms in the molecular function group (Table S4). The significantly enriched GO terms included metabolic process (Go: 0008152), cellular process (Go: 0009987) and cell (Go: 005623).

KEGG pathway enrichment analysis was performed to identify related metabolic pathways. The results showed that 48 metabolic pathways were significantly enriched by DEGs in 16 cross-combinations, 4–23 metabolic pathways were significantly enriched in each hybrid, and various metabolic pathways were significantly enriched in different cross-combinations (Table S5). There were 13 metabolic pathways in which DEGs were significantly enriched in 8 or more cross combinations. These metabolic pathways included photosynthesis (ko00195), photosynthesis–antenna proteins (ko00196), phenylalanine metabolism (ko00360), tryptophan metabolism (ko00380), carbon fixation in photosynthetic organisms (ko00710), limonene and pinene degradation (ko00903), sulfur metabolism (ko00920), stilbenoid, diarylheptanoid and gingerol biosynthesis (ko00945), metabolic pathways (ko01100), biosynthesis of secondary metabolites (ko01110), microbial metabolism in diverse environments (ko01120), plant–pathogen interaction (ko04626) and circadian rhythm–plants (ko04712).

### 2.5. MetabolicPathways Influencing the Degree of Heterosis

**Figure 3:**
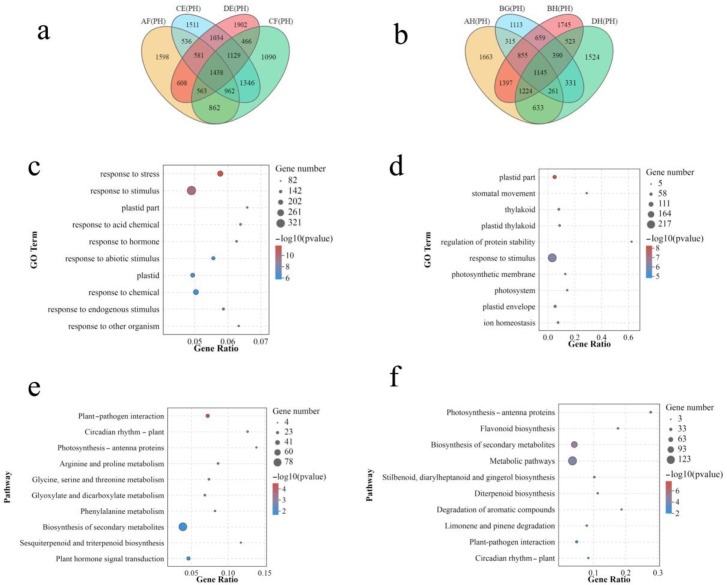
Analysis of genes related to heterosis. (**a**) Wien map of differentially expressed genes in strong heterosis hybrids. (**b**) Wien map of differentially expressed genes in weak heterosis hybrids. (**c**) GO enrichment of differentially expressed genes in strong heterosis hybrids. (**d**) GO enrichment analysis of differentially expressed genes in weak heterosis hybrids. (**e**) KEGG enrichment analysis of differentially expressed genes in strong heterosis hybrids. (**f**) KEGG enrichment analysis of differentially expressed genes in weak heterosis hybrids. The size of the bubble represents the number of genes contained in the pathway, and the color of the bubble represents the enrichment significance in the pathway.

In total, 1438commonDEGs in four strong heterosis hybrids (AF, CE, CF, DE)and 1145 common DEGs in four weak heterosis hybrids (AH, BG, BH, DH)were employed for enrichment analysis (Figure 3a,b). By enrichment analysis of the DEGs in four strong heterosis hybrids, it was found that the top 10 most enriched GO classifications belonged to the biological process classification and were shown in Figure 3c. The most dominant GO classification was response to stress (GO: 0006950). Go enrichment analysis of DEGs in four weak heterosis hybrids showed that six of the top ten GO classifications were related to cell component categorization, including plastid portion (GO: 0009579), thylakoid (GO: 0031976), photosynthetic membrane (GO: 0034357), photosystem (GO: 0009521), and plastid envelope (GO: 0009526) (Figure 3d).

The KEGG pathway enrichment analysis revealed that the DEGs in four strong heterosis hybrids were significantly enriched in plant pathogen interaction (ko04626) and plant circadian rhythm-plant (ko04712) (Figure 3e). The DEGs in four weak heterosis hybrids were significantly enriched in five pathways, which included photosynthesis-antenna proteins (ko00196), flavonoid biosynthesis (ko00941), metabolic pathways (ko01100), biosynthesis of secondary metabolites (ko01110), stilbenoid, diarylheptanoid and gingerol biosynthesis (ko00945) (Figure 3f).

### 2.6. Heterosis-Related Genes Found by WGCNA

**Figure 4:**
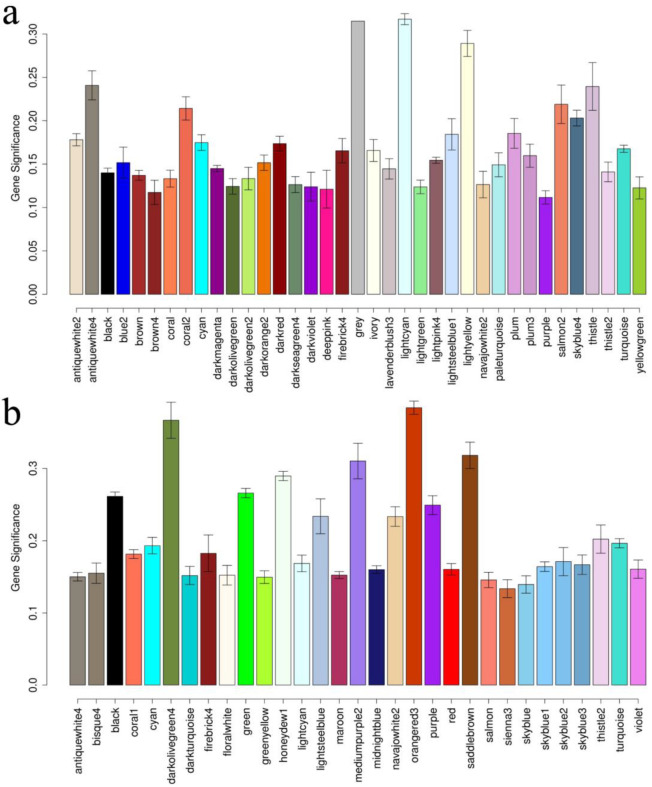
Relationship histogram between module and traits. (**a**): Relationship histogram between module and plant gross weight. (**b**): Relationship histogram between module and mid-parent valueof plant gross weight.

As shown in Figure 4a, WGCNA was used to seek expression data for all materials to identify PGW-related genes. A total of 37 modules, each represented by a distinct hue, were created from all the genes. A module (MM.lightcyan) was significantly correlated with PGW (Figure 4a). The genes in this module were significantly enriched in circadian rhythm-plant (ko04712), photosynthesis-antenna proteins (ko00196)and carotenoid biosynthesis (ko00906).

All genes were split into 31 modules, each represented by a distinct hue based on the mid-parent heterosis value (MPV) of the gene expression level and PGW. Among these modules, five modules (MM.mediumpurple2, MM.orangered3, MM.darkolivegreen4, MM.honeydew1 and MM.saddlebrow) showed a significant correlation with PGW (Figure 4b). The genes in the MM.mediumpurple2 module were significantly enriched in ribosome biogenesis in eukaryotes (ko03008) (Table S6). The genes in the MM.orangered3 module were significantly enriched in ribosome (ko03010) and ribosome biogenesis in eukaryotes (ko03008). The genes of the MM.honeydew1 module were significantly enriched in plant–pathogen interaction (ko04626) and glucosinolate biosynthesis (ko00966).

### 2.7. DEGs Related to Heterosis in Circadian Rhythm–Plant and Plant–Pathogen Interaction Pathway

**Figure 5:**
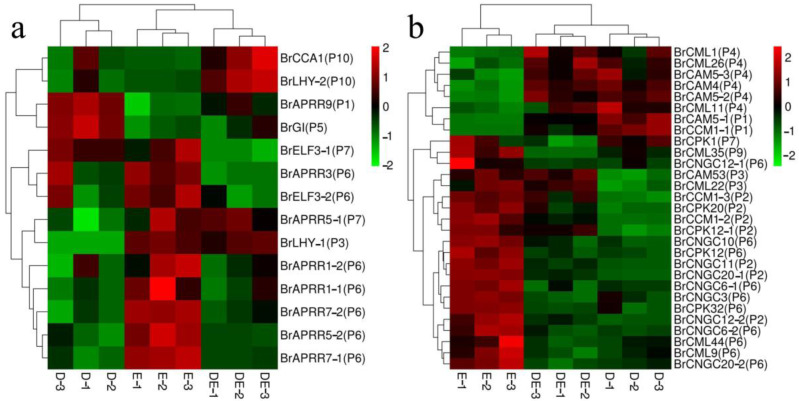
Heat map of differential expression genes related to heterosis. (**a**) The cluster heatmap of genes involving in circadian rhythm-plant pathway. (**b**) The cluster heatmap of genes regulating calcium. Green indicates the lowest expression. Red indicates the highest expression. The gene expression pattern is indicated in parentheses. D represents the female parent in the cross combination with strongest heterosis. E represents the male parent in the cross combination with strongest heterosis. DE represents the hybrid in the cross combination with strongest heterosis.

Based on the information above, one of the most important pathways for heterosis was the circadian rhythm-plant pathway. The genes in circadian rhythm-plant pathway showed varied expression patterns in the DE hybrid, which had the strongest heterosis of all cross combinations (Figure 5a). In the PRR protein family, *BrAPRR1-1*, *BrAPRR1-2*, *BrAPRR3*, *BrAPRR5-2*, *BrAPRR7-1* and *BrAPRR7-2* were showed a low parent-expression level, *BrAPRR5-1* was down-regulated in the manner of over-dominant expression, and *BrAPRR9* showedanadditive expression in the DE hybrid. In the manner of dominant expression, *BrCCA1* showed an up-regulation expression. In contrast, *BrLHY-2* was up-regulated in the manner of over-dominant expression. *BrLHY-1* showed a high parental expression level. As a result, compared to the high parent-expression level, most DEGs in the PRR protein family showed a lower expression level in DE hybrid. Instead, compared to the low parent-expression level, *BrCCA1*, *BrLHY-1*, and *BrLHY-2* conformed to higher expression levels in hybrid DE.

From the above, the plant–pathogen interaction pathway was related to heterosis. Genes controlling calcium displayed varied expression patterns in the DE hybrid, which had the strongest heterosis in all cross combinations (Figure 5b). In Calmodulin (*CaM*) and Calmodulin-like proteins (*CML*) genes, compared to the low parent-expression level, many genes had a higher expression level in hybrid DE, forexample, *BrCAM4*, *BrCAM5-2*, *BrCAM5-3*, *BrCML1*, *BrCML11*, *BrCML26*, *BrCAM53* and *BrCML22* showed a high parent-expression level, and *BrCAM5-1*, *BrCCM1-1*, *BrCCM1-2*, *BrCCM1-3* showed an additive expression. In the case of *CNGCs* (cyclic nucleotide-gated channels), *BrCNGC10*, *BrCNGC12-1*, *BrCNGC20-2*, *BrCNGC3*, *BrCNGC6-1*, *BrCNGC6-2* were showed a low parent-expression level, *BrCNGC11*, *BrCNGC12-2*, *BrCNGC20-1* showed an additive expression. For *CPKs* (calcium-dependent protein kinases), *BrCPK1* was down-regulated in the manner of over-dominant expression, *BrCPK12-1* and *BrCPK20* conformed to an additive expression, and *BrCPK12* and *BrCPK32* expressed a low parent-expression level. In sum, compared to the low parent-expression level, most genes of *CaM* and *CML*, showed a higher expression level in DE hybrid t. In contrast, compared to the high parent-expression level, most genes of *CNGCs* and *CPKs* exhibited a lower expression level in DE hybrid.

## 3. Discussion

Although heterosis has been successfully used from an agronomic standpoint in several crops, including hybrid rice and hybrid maize, the molecular mechanisms of heterosis still need to be fully understood. The mechanism of plant heterosis is still being studied, and most studies on the subject were conducted by a single or small number of special hybrids [35,36,37]. As a result, different hybrids have different reasons for heterosis, and it is uncertain whether there was any common variable contributing to heterosis. In this study, 16 hybrids created by 4 male parents and 4 female parents of Chinese cabbage were applied, allowing researchers to find the distinction between weak heterosis hybrids and strong heterosis hybrids.

In our study, DEGs were classified into additive, dominant and over-dominant expression pattern by comparing the gene expression level in hybrid and parents. Very few genes conformed to the additive and over-dominant expression pattern in Chinese cabbage, and most DEGs were expressed according to the dominant pattern. These results were distinct from other studies. The additive expression pattern of differentially expressed genes served as the primary expression pattern in maize [38]. Compared to other expression patterns, the number of genes with dominant expression pattern was considerably higher in tobacco. The number of genes with a male-dominant expression pattern was significantly greater than that of genes with a female-dominant expression pattern [39]. Ninety-five percent of the expressed genes in *Arabidopsis thaliana* were between the expression levels of the parents [27]. The main expression pattern of differentially expressed genes are distinct in different crops, consequently, the contributions of additive and dominant effect to heterosis are also distinct in different plants. The difference in gene expression pattern in different species may be due to differences in genetic background, classification and even environment, so the current explanation of the gene expression pattern in hybrids should be more reasonable from many aspects.

Identifying heterosis-related genes was an important target of the current study. Kong et al. found the high expression level of DEGs in photosynthesis pathway in hybrids depicting their relation with growth and hybrid vigor in Chinese cabbage [40]. Li et al. concluded that the expression levels of photosynthesis and chlorophyll synthesis-related differentially expressed genes were significantly different in the Chinese cabbage hybrid compared to the parental lines, resulting in increased photosynthesis capacity and chlorophyll content in the former [34]. In Pak choi hybrids, the increased photosynthetic activity was associated with an improved photosynthetic mechanism and larger leaves [41]. These results showed that photosynthesis is related to heterosis and were different from our conclusions. In our research, the photosynthesis (ko00195) and photosynthesis-antenna proteins (ko00196) were significantly enriched by DEGs in most cross combinations, however, these were not significantly enriched by DEGs in strong heterosis hybrids. Among them, the photosynthesis-antenna proteins were significantly enriched by DEGs in weak heterosis hybrids. Therefore, the photosynthesis is related to heterosis and may not make outstanding contributions to heterosis.

By KEGG pathway enrichment analysis, we discovered that 13 pathways, including plant–pathogen interaction (ko04626) and circadian rhythm-plant (ko04712), were significantly enriched by DEGs in the majority of cross combinations. Coincidentally, plant circadian rhythm pathways and plant–pathogen interaction were significantly enriched in the DEGs of strong heterosis hybrids. Therefore, the genes included in plant pathogen interaction and plant circadian rhythm pathways were related to heterosis and affected the degree of heterosis. WGCNA also proved a substantial relationship between the two pathways and PGW or PGW heterosis. Therefore, we hypothesized that plant pathogen interaction and plant circadian rhythm pathways have important contributions to heterosis.

The circadian rhythm clock’s impact on heterosis in hybrid crops has been proven. In *Arabidopsis*, hybrids and allopolyploids increased growth vigor and biomass by controlling physiological metabolic pathways that were mediated by circadian rhythm [42]. In maize, gene expression levels mediated by the circadian clock contributed to hybrid biomass [43]. Transcriptome analysis indicated that the circadian regulatory network may be related to heterosis in hybrid rice [34,44]. In addition, similar findings also have been reported in coffee, cotton, loquat and other plants [45,46]. In our work, the circadian rhythm clock core gene *BrCCA1*, *BrLHY-1*, and *BrLHY-2* conformed to a high parental expression level in a hybrid with the strongest heterosis. Compared to the high parent-expression level, most DEGs of the PRR protein family showed lower expression levels. Our results suggested that genes in the circadian rhythm pathway may be related to heterosis. However, since there is only experimental evidence in the morning, more research is required to determine how these genes influence heterosis.

Calcium, an essential secondary messenger in eukaryotic cells, plays major roles in plant growth and development [47,48]. There have been some reports about the relationship between calcium and heterosis. By proteomic analysis, calmodulin-binding transcription activators were detected in hybrid rice under heat stress [49]. Compared with parents, the calmodulin binding protein was differentially expressed in a soybean hybrid (Jilin 38 × EXP) [50]. In our study, although most genes in *CaM* and *CML* had a high parental expression level, most genes in *CPK* and *CNGC* had lower expression level than high parent-expression level in hybrid with strongest heterosis. However, how these genes act on heterosis still needs to be further explored.

## 4. Materials and Methods

### 4.1. Plant Materials

Eight inbred lines and sixteen hybrids were used for heterosis analysis (Table 3). All materials were developed and provided by the Chinese cabbage research group, at the College of Horticulture, Northwest A&F University, Yangling, China. Eight Chinese cabbage inbred lines were self-bred for at least eight generations. The inbred line parents were used for artificial cross-pollination to obtain the hybrids by in complete diallel crossing design. In all the materials, inbred lines A(original name: S93), B(original name: S129), C(original name: S96), D(original name: S256), were female parents, inbred lines E(original name: S602), F(original name: S1063), G(original name: S568), H(original name: S346) were male parents, and the other materials were hybrids.

The experiment was conducted at Yangling Wuquan test field in Shaanxi, China. At the middle stage of heading (about 70 days), the first outer leaf from top to bottom was collected with the help of sterile scissors at 9:30–10:30 am. The samples were wrapped in tin foil, quickly frozen with liquid nitrogen, stored at −80 °C, and used for RNA-Seq. Specimens from three individuals were mixed as one test sample, and three replicates were taken from each material. At maturity (about 100 days), the PGW was investigated in parents and hybrids. The PGW data were described in detail in a previous study [51].

The code in the first column represents the female parent, the code in the first line represents the male parent, and the rests are the corresponding hybrids.

The MPV was calculated for PGW according to the formula:

(1) $$\mathrm{MPV}\;=\frac{F1-\mathrm{MP}}{\mathrm{MP}}*100\%$$

where F_1_ is the value of hybrid, MP is the mean value of two parents. Then, these hybrids were separated into three groups according to the MPV of PGW (Table 4). When the MPV of PGW is higher than 140, the hybrid belongs to a strong heterosis combination, including AF, CE, DE, and CF hybrid. When the MPV of PGW is lower than 40, the hybrid belongs toa weak heterosis combination, including AH, BG, BH, and DH hybrid. When the MPV of PGW is between 40 and 140, the hybrid was in the moderate heterosis group (The details were obtained from previous project) [51].

### 4.2. RNA Isolation, cDNA Library Construction, and RNA-Seq

Following the manufacturer’s instructions, RNA was extracted using the Trizol reagent (Invitrogen, Carlsbad, CA, USA) before the decontamination of genomic DNA using DNaseI (TaKaRa, Otsu, Japan). A NanoDrop 8000 spectrophotometer (Thermo Scientific, Waltham, MA, USA), an Agilent 2100 Bioanalyzer (Agilent Technologies, Santa Clara, CA, USA), and 1.0% agarose gels were used to evaluate the quality, purity, and integrity of the RNA.

Total RNA was isolated from the sample, followed by the enrichment of mRNA using Oligo (dT) magnetic beads, shortening of the acquired mRNA by adding a fragmentation buffer, and using the short fragmented mRNA as a template. The first strand of cDNA was created using six-base random primers (random hexamers), whereas the second strand was created by adding buffer, dNTPs, RNase H, and DNA polymerase I. Then, the cDNA fragments were purified with aQiaQuick PCR extraction kit, end-repaired, poly(A) added, and ligated to Illumina sequencing adapters. The ligation products were size selected by agarose gel electrophoresis, PCR-amplified, and sequenced using Illumina HiSeq_TM_ by Genedenovo Biotechnology Co., Ltd. (Guangzhou, China). The obtained raw data from constructed cDNA libraries were deposited in NCBI Sequence Read Archive (SRA, http://www.ncbi.nlm.nih.gov/Traces/sra/ (accessed on 1 June 2022)) under the accession number: BioProject PRJNA876066 (The details were obtained from previous project) [51].

### 4.3. Identification of Differentially Expressed Genes

After the total RNA was extracted from the sample, the constructed library was sequenced with Illumina HiSeq^TM^. After filtered, the reads were mapped to the reference genome, and the transcripts were assembled using cuff links to obtain the known transcripts and new transcripts. The mean FPKM value was taken for each gene in three biological repeats. DEGs between hybrid and parents were identified for each crossing combination using the DESeq2 package in R (false discovery rate adjusted *p* value < 0.01).

### 4.4. Analysis Expression Pattern of Differentially Expressed Genes

DEGs were divided into 12 expression patterns using R language (Table 5). When the DEGs met all screening conditions, it was considered to belong to the corresponding expression pattern. P1 and P2 were additive expression. P3–P6 conformed to dominant expression. P7–P12 were over-dominant expression.

### 4.5. Functional Enrichment Analysis

To identify possible biological functions, Gene Ontology (GO) and Kyoto Encyclopedia of Genes and Genomes (KEGG) annotations were performed for DEGs. The DEGs were mapped to GO database (http://www.geneontology.org/) (accessed on 1 March 2021) using GOseq R package, and signifcantly enriched GO terms were identified if an adjusted *p* < 0.05. The KEGG pathways were assigned using the KEGG software package (http://www.kegg.jp/) (accessed on 1 March 2021), and considered significant if an adjusted *p* < 0.05.

### 4.6. Weighted Gene Coexpression Network Analysis

Gene co-expression modules were assigned using the weighted gene coexpression network analysis (WGCNA) R package. The minimum module size was 50 genes and the soft threshold power β was set to 11. Significant module–trait associations were identified by correlating the module eigengenes with the measured value or MPV of PGW. The eigengenes represented the gene expression pattern within a module. A module was considered significant if the *p* < 0.05.

## 5. Conclusions

We concluded that the dominant expression pattern is the main expression pattern in Chinese cabbage hybrids. The genes in the plant–pathogen interaction and circadian rhythm-plant pathway were related to heterosis in Chinese cabbage. However, it is needs to be further explored.

## Figures and Tables

**Table 1:** Quantity statistics of differentially expressed genes.

Group	Female Parent vs. Male Parent	Female Parent vs. Hybrid	Male Parent vs. Hybrid	DEGs Ratio
A-AE-E	10,252	2841	6374	65.23
A-AF-F	7203	3813	4719	73.14
A-AG-G	8643	3141	4098	60.44
A-AH-H	9951	3578	5211	62.96
B-BE-E	10,043	3412	5829	66.45
B-BF-F	7053	2349	5136	70.81
B-BG-G	8364	2554	3442	54.50
B-BH-H	10,215	3004	6224	66.90
C-CE-E	8078	5990	4424	74.85
C-CF-F	6580	5533	3872	72.14
C-CG-G	6044	4667	2312	63.78
C-CH-H	7880	5095	7036	76.79
D-DE-E	9064	3009	6376	68.53
D-DF-F	5815	1796	2244	51.93
D-DG-G	7853	3313	2822	55.52
D-DH-H	7462	3495	3550	64.84

DEGs Ratio: the ratio of DEGs (there were differential expression in two comparison groups (female parent vs. hybrid or male parent vs. hybrid)) to DEGs (there were differential expression in a comparison group (female parent-vs-male parent)).

**Table 2:** A number of genes in each of the 12 expression patterns of differentially expressed genes.

Group	The Number of Genes											
	P1	P2	P3	P4	P5	P6	P7	P8	P9	P10	P11	P12
AE	575	689	806	2139	385	2039	75	4	15	77	15	19
AF	519	427	951	1234	638	1394	233	3	75	99	21	6
AG	386	476	1177	1192	539	1412	60	4	12	116	14	12
AH	477	481	1302	1781	675	1453	67	12	12	174	22	50
BE	771	700	1166	2289	360	1342	28	4	6	90	21	15
BF	399	327	504	1789	312	1613	59	2	24	199	15	9
BG	428	450	996	1162	412	1101	17	1	0	23	1	7
BH	548	468	992	2703	525	1522	71	4	12	127	9	51
CE	376	405	1726	964	1538	801	257	47	15	602	50	124
CF	180	230	1089	712	1399	986	503	22	67	485	30	32
CG	102	132	1510	470	1046	477	255	42	6	380	9	61
CH	196	192	1531	1047	1242	595	292	145	19	524	35	208
DE	623	728	736	1753	342	1966	127	4	28	122	25	7
DF	209	175	881	903	166	667	8	2	4	80	8	5
DG	253	371	1464	910	541	794	28	2	3	116	9	12
DH	366	380	1462	1203	591	774	59	7	9	147	10	36
Average	401	414	1143	1391	669	1184	134	19	19	210	18	41
Standard error	44.18	44.16	81.42	150.00	99.79	116.28	33.16	8.83	5.23	43.78	2.98	13.13

P1 and P2 conform to an additive expression pattern. P3, P4, P5 and P6 conform toa dominant expression pattern. P7, P8, P9, P10, P11 and P12 conform to an over-dominant expression pattern.

**Table 3:** The code of inbred lines and hybrids of Chinese cabbage.

	♂	E	F	G	H
♀					
A		AE	AF	AG	AH
B		BE	BF	BG	BH
C		CE	CF	CG	CH
D		DE	DF	DG	DH

**Table 4:** Mid-parent heterosis value analysis of plant gross weight in hybrids.

Hybrids Codes	Heterosis Level	Mid-Parent Heterosis
AE	middle	87.25
AF	strong	227.83
AG	middle	72.65
AH	weak	25.51
BE	middle	84.36
BF	middle	112.74
BG	weak	28.63
BH	weak	15.69
CE	strong	141.44
CF	strong	146.32
CG	middle	105.44
CH	middle	97.09
DE	strong	233.98
DF	middle	121.61
DG	middle	62.37
DH	weak	30.09

**Table 5:** Screening conditions for different expression patterns.

Expression Patterns	Screening Conditions
P1	a: FPKM_F_ > FPKM_M_ b: FPKM_M_ < FPKM_H_ < FPKM_F_
P2	a: FPKM_F_ < FPKM_M_ b: FPKM_F_ < FPKM_H_ < FPKM_M_
P3	a: FPKM_F_ < FPKM_M_ b: FPKM_H_ = FPKM_M_
P4	a: FPKM_F_ > FPKM_M_ b: FPKM_H_ = FPKM_M_
P5	a: FPKM_F_ > FPKM_M_ b: FPKM_H_ = FPKM_F_
P6	a: FPKM_F_ < FPKM_M_ b: FPKM_H_ = FPKM_F_
P7	a: FPKM_F_ = FPKM_M_ b: FPKM_H_ < FPKM_F_
P8	a: FPKM_F_ > FPKM_M_ b: FPKM_H_ < FPKM_F_ c: FPKM_H_ < FPKM_M_
P9	a: FPKM_F_ < FPKM_M_ b: FPKM_H_ < FPKM_F_ c: FPKM_H_ < FPKM_M_
P10	a: FPKM_F_ = FPKM_M_ b: FPKM_H_ > FPKM_F_ c: FPKM_H_ > FPKM_M_
P11	a: FPKM_F_ > FPKM_M_ b: FPKM_H_ > FPKM_F_ c: FPKM_H_ > FPKM_M_
P12	a: FPKM_F_ < FPKM_M_ b: FPKM_H_ > FPKM_F_ c: FPKM_H_ > FPKM_M_

FPKM_F_ represents the FPKM in female parent; FPKM_H_ represents the FPKM in hybrid; FPKM_M_ represents the FPKM in male parent. “=”means that there was no difference between groups.

## Data Availability

The RNA-seq data have been deposited with the NCBI with the dataset identifier PRJNA876066.

## Supplementary Materials

The following are available online at https://www.mdpi.com/article/10.3390/plants12051195/s1, Table S1: Overview of transcriptome sequencing data, Table S2: Assembling overview of transcriptome sequencing data, Table S3: The genes number of transcriptome sequencing, Table S4: Go term classification table of differential expression genes in different cross combinations, Table S5: KEGG enrichment analysis of differential expression genes in different cross combinations, Table S6: KEGG Enrichment analysis of module genes in hybrids of Chinese cabbage.
