# Supplementary material for: Transcriptome Analysis Reveals a Major Gene Expression Pattern and Important Metabolic Pathways in the Control of Heterosis in Chinese Cabbage
Source: Plants (Basel). 2023 Mar 6;12(5):1195. doi: 10.3390/plants12051195 (PMC10005390; doi:10.3390/plants12051195)
Supplement: Supplementary file 1 [file plants-12-01195-s001.zip › plants-2204414-supplementary.pdf]

Table S1. Overview of transcriptome sequencing data.

Sample	Clean Data(bp)	HQ Clean Data(bp)	Q20(%)	Q30(%)	N (%)	GC(%)
A-1	6594998400	6422389482	98.96%	96.19%	0.00%	48.67%
A-2	10317542100	10054023418	98.91%	96.11%	0.00%	48.67%
A-3	8945708700	8711767639	98.88%	95.97%	0.00%	48.69%
B-1	10255316700	9985192793	98.90%	96.07%	0.00%	48.51%
B-2	10138459500	9852970551	98.83%	95.85%	0.00%	48.81%
B-3	8347193700	8132633044	98.97%	96.20%	0.00%	48.60%
C-1	10439619600	10146781386	99.05%	96.44%	0.00%	48.78%
C-2	10999920000	10702287570	98.93%	96.09%	0.00%	48.65%
C-3	6615540900	6422074745	98.92%	96.00%	0.00%	48.78%
D-1	16253250900	15775601786	98.89%	96.04%	0.00%	48.95%
D-2	12939807000	12570537440	99.01%	96.36%	0.00%	48.89%
D-3	14455863600	14053223728	99.06%	96.49%	0.00%	48.84%
E-1	10637226900	10298788888	98.81%	95.80%	0.00%	48.77%
E-2	10326297600	10057728210	99.08%	96.53%	0.00%	48.70%
E-3	9830315700	9568748224	99.06%	96.48%	0.00%	48.74%
F-1	12346165800	12008333348	99.01%	96.37%	0.00%	48.93%
F-2	14678412900	14203535913	98.90%	96.04%	0.00%	48.88%
F-3	11929335300	11576750706	98.98%	96.24%	0.00%	48.77%
G-1	8143155000	7919152251	98.97%	96.21%	0.00%	48.76%
G-2	14202638700	13829082837	99.04%	96.42%	0.00%	48.73%
G-3	9658517700	9374778566	98.95%	96.19%	0.00%	48.64%
H-1	8839325100	8578228445	98.99%	96.28%	0.00%	48.84%
H-2	10485706500	10205946935	99.04%	96.44%	0.00%	48.84%
H-3	15260682000	14838687481	99.02%	96.38%	0.00%	48.80%
AE-1	10471353300	10178973748	98.94%	96.18%	0.00%	48.68%
AE-2	7482231600	7264454324	98.92%	96.14%	0.00%	48.67%
AE-3	7688638500	7477515396	98.99%	96.30%	0.00%	48.71%
AF-1	11343029100	11007909936	98.89%	96.05%	0.00%	48.83%
AF-2	10074441600	9786393479	99.02%	96.39%	0.00%	48.79%
AF-3	12963968400	12609362873	99.02%	96.39%	0.00%	48.78%
AG-1	8603392200	8337234064	98.93%	96.13%	0.00%	48.76%
AG-2	10260536400	9931134676	99.00%	96.33%	0.00%	48.74%
AG-3	6883426500	6657738686	99.02%	96.44%	0.00%	48.69%
AH-1	7993327200	7763353570	99.09%	96.59%	0.00%	48.67%
AH-2	14084336700	13744446536	99.08%	96.54%	0.00%	48.73%
AH-3	11970223500	11680269569	99.07%	96.53%	0.00%	48.55%
BE-1	11789282700	11508003025	98.93%	96.09%	0.00%	48.51%
BE-2	10130693700	9884338027	99.00%	96.32%	0.00%	48.56%
BE-3	10648883700	10391178570	99.03%	96.38%	0.00%	48.57%
BF-1	10779615000	10525008250	99.12%	96.65%	0.00%	48.50%
BF-2	14108965800	13748325316	98.98%	96.23%	0.00%	48.60%
BF-3	11211783600	10946952969	99.08%	96.53%	0.00%	48.74%
BG-1	8465539800	8239268192	98.99%	96.29%	0.00%	48.67%
BG-2	10531306800	10265042769	99.06%	96.50%	0.00%	48.58%
BG-3	10529558100	10267823172	99.04%	96.41%	0.00%	48.57%
BH-1	10399698300	10131823965	99.06%	96.48%	0.00%	48.53%
BH-2	9769117200	9536155293	98.99%	96.27%	0.00%	48.66%

Sample	Clean Data(bp)	HQ Clean Data(bp)	Q20(%)	Q30(%)	N (%)	GC(%)
BH-3	6309939900	6157709066	98.99%	96.27%	0.00%	48.43%
CE-1	11303251500	11024628899	98.93%	96.07%	0.00%	48.54%
CE-2	11685800400	11411147710	98.94%	96.11%	0.00%	48.53%
CE-3	8658100800	8434209017	98.96%	96.18%	0.00%	48.68%
CF-1	10006513500	9748986976	98.91%	96.02%	0.00%	48.73%
CF-2	5999270700	5836013171	98.98%	96.25%	0.00%	48.84%
CF-3	11417490300	11136884193	99.04%	96.41%	0.00%	48.96%
CG-1	7363614300	7186954560	99.02%	96.36%	0.00%	48.65%
CG-2	10916415600	10648720629	98.99%	96.28%	0.00%	48.47%
CG-3	9650770500	9384160349	98.74%	95.76%	0.00%	48.87%
CH-1	11087527500	10825831949	98.93%	96.07%	0.00%	48.78%
CH-2	7875193200	7676861386	98.90%	96.00%	0.00%	48.70%
CH-3	8920348200	8699924078	99.02%	96.36%	0.00%	48.52%
DE-1	7741184700	7547807258	98.90%	96.02%	0.00%	48.80%
DE-2	7391739600	7199819507	98.98%	96.25%	0.00%	48.88%
DE-3	10836456300	10582343002	99.06%	96.48%	0.00%	48.98%
DF-1	8932945800	8701620536	98.97%	96.22%	0.00%	48.70%
DF-2	10359043200	10098122692	99.01%	96.32%	0.00%	48.69%
DF-3	9655847100	9419629694	99.03%	96.38%	0.00%	48.92%
DG-1	9394145700	9153075163	99.00%	96.31%	0.00%	48.58%
DG-2	13119792000	12796108687	98.96%	96.17%	0.00%	48.79%
DG-3	8712075300	8488210555	99.02%	96.36%	0.00%	48.68%
DH-1	9470340300	9214524723	98.85%	95.87%	0.00%	48.74%
DH-2	9853066800	9585657189	98.98%	96.24%	0.00%	48.81%
DH-3	10484229900	10203969247	98.96%	96.19%	0.00%	48.77%

Table S2. Assembling overview of transcriptome sequencing data.

Sample	Repetition	Total Reads	Mapping Ratio	Unique Mapped Ratio
A	1	42121426	76.01%	74.12%
A	2	66131928	76.70%	74.70%
A	3	56982496	76.44%	74.53%
B	1	65520952	79.81%	77.66%
B	2	61915878	79.82%	77.71%
B	3	52570664	80.01%	77.87%
C	1	65977452	77.17%	75.10%
C	2	70735350	77.22%	75.16%
C	3	41843442	77.10%	75.14%
D	1	102283768	77.39%	75.43%
D	2	82294506	77.51%	75.54%
D	3	93006494	77.45%	75.46%
E	1	67845388	75.95%	74.06%
E	2	66325748	76.13%	74.19%
E	3	63199992	76.27%	74.35%
F	1	79340748	77.71%	75.60%
F	2	92191874	77.10%	75.06%
F	3	76458786	76.98%	74.96%
G	1	52167178	76.52%	74.49%
G	2	90794486	76.48%	74.36%
G	3	62069814	76.55%	74.52%
H	1	56947004	76.34%	74.20%
H	2	67357370	77.48%	75.47%
H	3	97847780	77.33%	75.26%
AE	1	66603362	76.82%	74.97%
AE	2	48040630	76.70%	74.87%
AE	3	49022764	76.72%	74.90%
AF	1	72610522	76.99%	75.11%
AF	2	64477432	77.60%	75.75%
AF	3	82860794	77.37%	75.42%
AG	1	54715618	76.79%	74.88%
AG	2	65638400	76.83%	74.91%
AG	3	44170326	76.93%	75.07%
AH	1	50898758	77.42%	75.51%
AH	2	90777848	77.26%	75.26%
AH	3	76734988	77.58%	75.57%
BE	1	74651376	77.93%	75.98%
BE	2	64355092	78.15%	76.20%
BE	3	67818020	77.93%	76.02%
BF	1	68950490	79.23%	77.28%
BF	2	89907180	78.60%	76.66%

Sample	Repetition	Total Reads	Mapping Ratio	Unique Mapped Ratio
BF	3	71059046	78.77%	76.77%
BG	1	53629716	78.73%	76.77%
BG	2	67030902	79.08%	77.10%
BG	3	66958358	78.66%	76.65%
BH	1	66663318	78.73%	76.70%
BH	2	61764302	78.44%	76.41%
BH	3	40426558	78.92%	76.92%
CE	1	72809350	76.03%	74.24%
CE	2	75326652	76.18%	74.34%
CE	3	54992942	75.90%	74.14%
CF	1	63998882	76.34%	74.34%
CF	2	37808384	77.03%	75.21%
CF	3	71524462	76.88%	74.98%
CG	1	47116010	76.51%	74.57%
CG	2	70180226	76.83%	74.88%
CG	3	60476380	75.62%	73.72%
CH	1	68791064	76.54%	74.66%
CH	2	50443988	76.55%	74.66%
CH	3	57487742	76.87%	74.96%
DE	1	49466066	77.18%	75.37%
DE	2	46491060	77.28%	75.49%
DE	3	67633820	77.49%	75.67%
DF	1	56866306	77.69%	75.80%
DF	2	66433156	77.72%	75.66%
DF	3	61312746	77.62%	75.75%
DG	1	59719070	77.41%	75.49%
DG	2	83947824	76.98%	75.04%
DG	3	55022884	77.08%	75.19%
DH	1	60595358	77.58%	75.61%
DH	2	61889238	77.73%	75.78%
DH	3	65897584	77.53%	75.58%

Table S3. The genes number of transcriptome sequencing.

Sample Name	Repetition	Known Gene Num	New Gene Num	All Gene Num
A	1	24927 (54.67%)	1339	26266
A	2	25811 (56.61%)	1353	27164
A	3	25944 (56.90%)	1407	27351
A	summary	28234 (61.92%)	1534	29768
B	1	25855 (56.71%)	1404	27259
B	2	25764 (56.51%)	1421	27185
B	3	25748 (56.47%)	1405	27153
B	summary	28211 (61.87%)	1524	29735
C	1	26271 (57.62%)	1459	27730
C	2	26130 (57.31%)	1476	27606
C	3	25134 (55.12%)	1384	26518
C	summary	28409 (62.31%)	1583	29992
D	1	26909 (59.02%)	1452	28361
D	2	26655 (58.46%)	1457	28112
D	3	27128 (59.50%)	1482	28610
D	summary	29255 (64.16%)	1591	30846
E	1	26709 (58.58%)	1410	28119
E	2	26499 (58.12%)	1371	27870
E	3	26488 (58.09%)	1393	27881
E	summary	29256 (64.16%)	1517	30773
F	1	27051 (59.33%)	1479	28530
F	2	27569 (60.46%)	1526	29095
F	3	27093 (59.42%)	1518	28611
F	summary	29915 (65.61%)	1654	31569
G	1	26969 (59.15%)	1552	28521
G	2	28222 (61.90%)	1627	29849
G	3	27471 (60.25%)	1599	29070
G	summary	30615 (67.15%)	1723	32338
H	1	26333 (57.75%)	1486	27819
H	2	26511 (58.14%)	1525	28036
H	3	27455 (60.21%)	1572	29027
H	summary	29304 (64.27%)	1666	30970
AE	1	26985 (59.18%)	1546	28531
AE	2	26008 (57.04%)	1481	27489
AE	3	26323 (57.73%)	1544	27867
AE	summary	29262 (64.18%)	1662	30924
AF	1	26934 (59.07%)	1585	28519
AF	2	27090 (59.41%)	1565	28655
AF	3	27397 (60.09%)	1621	29018
AF	summary	29802 (65.36%)	1739	31541
AG	1	26928 (59.06%)	1588	28516

Sample Name	Repetition	Known Gene Num	New Gene Num	All Gene Num
AG	2	26921 (59.04%)	1571	28492
AG	3	25920 (56.85%)	1496	27416
AG	summary	29348 (64.37%)	1709	31057
AH	1	26454 (58.02%)	1534	27988
AH	2	27224 (59.71%)	1571	28795
AH	3	27253 (59.77%)	1561	28814
AH	summary	29538 (64.78%)	1686	31224
BE	1	27646 (60.63%)	1593	29239
BE	2	27502 (60.32%)	1579	29081
BE	3	27495 (60.30%)	1571	29066
BE	summary	30259 (66.36%)	1697	31956
BF	1	26726 (58.62%)	1594	28320
BF	2	27485 (60.28%)	1638	29123
BF	3	26841 (58.87%)	1601	28442
BF	summary	29518 (64.74%)	1733	31251
BG	1	26720 (58.60%)	1612	28332
BG	2	27321 (59.92%)	1621	28942
BG	3	26844 (58.87%)	1623	28467
BG	summary	29653 (65.04%)	1737	31390
BH	1	26534 (58.19%)	1535	28069
BH	2	26510 (58.14%)	1544	28054
BH	3	25505 (55.94%)	1482	26987
BH	summary	28740 (63.03%)	1646	30386
CE	1	27673 (60.69%)	1543	29216
CE	2	27340 (59.96%)	1561	28901
CE	3	27008 (59.23%)	1524	28532
CE	summary	30119 (66.06%)	1666	31785
CF	1	26563 (58.26%)	1551	28114
CF	2	25680 (56.32%)	1549	27229
CF	3	26768 (58.71%)	1553	28321
CF	summary	29313 (64.29%)	1739	31052
CG	1	26350 (57.79%)	1583	27933
CG	2	27725 (60.81%)	1621	29346
CG	3	26557 (58.25%)	1570	28127
CG	summary	29883 (65.54%)	1748	31631
CH	1	27058 (59.34%)	1543	28601
CH	2	26308 (57.70%)	1504	27812
CH	3	26571 (58.28%)	1519	28090
CH	summary	29442 (64.57%)	1673	31115
DE	1	26347 (57.78%)	1517	27864
DE	2	26253 (57.58%)	1514	27767
DE	3	26991 (59.20%)	1568	28559

Sample Name	Repetition	Known Gene Num	New Gene Num	All Gene Num
DE	summary	29200 (64.04%)	1674	30874
DF	1	26623 (58.39%)	1590	28213
DF	2	27053 (59.33%)	1604	28657
DF	3	26503 (58.13%)	1540	28043
DF	summary	29488 (64.67%)	1735	31223
DG	1	27009 (59.24%)	1622	28631
DG	2	27453 (60.21%)	1627	29080
DG	3	26650 (58.45%)	1604	28254
DG	summary	29627 (64.98%)	1742	31369
DH	1	26673 (58.50%)	1564	28237
DH	2	26812 (58.80%)	1571	28383
DH	3	27090 (59.41%)	1584	28674
DH	summary	29393 (64.47%)	1702	31095

Table S4. Go term classification table of differential expression genes in different cross combinations.

GO Term (level2)	GO Term (level1)	GO ID (level2)	Number of Genes															
			AE	AF	AG	AH	BE	BF	BG	BH	CE	CF	CG	CH	DE	DF	DG	DH
reproduction	Biological Process	GO:0000003	265	213	225	278	266	242	165	306	305	254	207	400	287	219	185	223
cell killing	Biological Process	GO:0001906	1	2	1				1		1	1	1	3	4	2	2	2
immune system process	Biological Process	GO:0002376	157	194	123	135	166	150	123	183	149	191	106	219	187	140	88	94
behavior	Biological Process	GO:0007610				1	1			1			1	2		1		1
metabolic process	Biological Process	GO:0008152	2050	1887	1547	1903	2012	1692	1259	2129	2182	2032	1479	2570	2130	1537	1314	1508
cellular process	Biological Process	GO:0009987	2192	2083	1704	2134	2191	1820	1380	2304	2360	2172	1537	2759	2262	1617	1376	1602
reproductive process	Biological Process	GO:0022414	263	209	221	274	262	233	161	300	299	247	200	394	283	215	183	222
biological adhesion	Biological Process	GO:0022610	11	11	13	14	15	9	12	11	16	18	6	19	15	15	8	11
signaling	Biological Process	GO:0023052	390	443	319	387	387	338	239	402	386	424	249	494	419	272	194	268
multicellular organismal process	Biological Process	GO:0032501	449	412	359	440	431	374	264	484	504	454	314	577	461	323	266	324
developmental	Biological	GO:0032	665	574	537	646	632	536	390	685	706	628	467	852	679	456	418	492

GO Term (level2)	GO Term (level1)	GO ID (level2)	Number of Genes															
			AE	AF	AG	AH	BE	BF	BG	BH	CE	CF	CG	CH	DE	DF	DG	DH
process	Process	502																
growth	Biological Process	GO:0040007	126	116	106	121	116	105	69	132	122	114	67	141	121	83	67	84
locomotion	Biological Process	GO:0040011	5	4	3	5	5	3	2	6	3	2	3	4	7	5	2	6
single-organism process	Biological Process	GO:0044699	1978	1863	1550	1896	1942	1632	1242	2040	2125	1953	1370	2456	2030	1483	1220	1433
rhythmic process	Biological Process	GO:0048511	42	47	41	47	43	34	26	42	50	34	30	57	50	30	23	40
response to stimulus	Biological Process	GO:0050896	1490	1467	1189	1443	1487	1250	939	1531	1576	1534	1038	1833	1536	1086	872	1070
localization	Biological Process	GO:0051179	716	709	550	695	735	613	439	775	769	739	461	905	762	537	437	523
multi-organism process	Biological Process	GO:0051704	357	390	274	311	335	313	244	389	351	417	245	452	391	281	199	247
biological regulation	Biological Process	GO:0065007	1199	1175	930	1133	1179	985	721	1241	1234	1163	761	1461	1185	840	645	803
cellular component organization or biogenesis	Biological Process	GO:0071840	675	577	527	657	686	569	429	720	749	661	488	851	710	468	464	509
detoxification	Biological Process	GO:0098754	3	2	2	2	2	3	1	3	4	3	3	4	1	1	3	2
extracellular	Cellular	GO:0005	130	124	111	125	134	125	90	154	142	145	103	155	155	99	103	114

GO Term (level2)	GO Term (level1)	GO ID (level2)	Number of Genes															
			AE	AF	AG	AH	BE	BF	BG	BH	CE	CF	CG	CH	DE	DF	DG	DH
region	Component	576																
cell	Cellular Component	GO:0005623	2630	2416	2068	2607	2612	2201	1617	2819	2854	2575	1904	3369	2661	1955	1673	1946
nucleoid	Cellular Component	GO:0009295		1	2	3	2	2	2	2	1	1	1	2	1	1	2	2
membrane	Cellular Component	GO:0016020	1094	1002	801	1013	1067	845	668	1104	1152	1031	715	1310	1097	771	611	767
virion	Cellular Component	GO:0019012	2	2	2	3	3	1	2	2	2		1	2	3	1	1	2
cell junction	Cellular Component	GO:0030054	237	217	181	209	232	203	150	243	255	241	175	282	239	162	164	182
extracellular matrix	Cellular Component	GO:0031012	10	7	7	7	8	11	2	9	7	11	5	10	9	11	8	9
membrane-enclosed lumen	Cellular Component	GO:0031974	21	18	17	18	22	20	16	28	23	19	18	34	31	21	19	22
macromolecular complex	Cellular Component	GO:0032991	259	214	179	222	281	240	189	323	326	286	267	388	297	211	225	229
organelle	Cellular Component	GO:0043226	2288	2094	1794	2245	2261	1919	1417	2484	2484	2220	1645	2945	2334	1701	1462	1708
extracellular matrix component	Cellular Component	GO:0044420	1	1	3	2		2	1	3	1	2	2	2	1	2	1	1
extracellular region part	Cellular Component	GO:0044421	8	7	6	9	4	7	3	8	8	8	6	9	5	5	9	2
organelle part	Cellular	GO:0044	678	619	516	649	710	588	485	828	789	680	551	909	807	519	506	580

GO Term (level2)	GO Term (level1)	GO ID (level2)	Number of Genes															
			AE	AF	AG	AH	BE	BF	BG	BH	CE	CF	CG	CH	DE	DF	DG	DH
	Component	422																
virion part	Cellular Component	GO:0044423	2	2	2	3	3	1	2	2	2		1	2	3	1	1	2
membrane part	Cellular Component	GO:0044425	600	537	445	561	555	471	364	602	604	549	386	687	596	431	303	414
cell part	Cellular Component	GO:0044464	2626	2415	2067	2605	2609	2201	1616	2816	2852	2572	1903	3366	2660	1954	1670	1945
supramolecular fiber	Cellular Component	GO:0099512	4	7	4	4	4	3	4	5	4	6	4	6	4	7	5	4
transcription factor activity, protein binding	Molecular Function	GO:0000988	8	7	9	7	9	8	4	8	9	6	7	11	7	4	3	5
nucleic acid binding transcription factor activity	Molecular Function	GO:0001071	262	271	235	273	240	227	160	260	258	247	167	329	227	179	127	177
catalytic activity	Molecular Function	GO:0003824	1415	1292	1068	1311	1343	1083	814	1376	1460	1360	954	1711	1427	981	839	972
signal transducer activity	Molecular Function	GO:0004871	27	26	23	31	28	16	16	23	22	19	18	29	26	15	13	17
structural molecule activity	Molecular Function	GO:0005198	55	49	51	51	59	65	52	79	85	77	100	91	81	44	104	69
transporter activity	Molecular Function	GO:0005215	196	195	156	196	213	167	134	231	219	212	127	242	209	165	126	159

GO Term (level2)	GO Term (level1)	GO ID (level2)	Number of Genes															
			AE	AF	AG	AH	BE	BF	BG	BH	CE	CF	CG	CH	DE	DF	DG	DH
binding	Molecular Function	GO:0005488	1617	1515	1283	1621	1570	1343	986	1707	1732	1570	1111	2025	1592	1166	942	1163
electron carrier activity	Molecular Function	GO:0009055	1	1	1	1	3	1	1	2	1			1	2		2	1
antioxidant activity	Molecular Function	GO:0016209	21	26	25	26	29	20	29	29	29	30	17	33	29	20	29	25
translation regulator activity	Molecular Function	GO:0045182	2	2	2	2	4	2	1	4	1	1	1	3	1	1	1	1
molecular transducer activity	Molecular Function	GO:0060089	33	31	20	30	28	21	12	25	25	21	14	30	25	21	13	25
molecular function regulator	Molecular Function	GO:0098772	32	24	21	34	27	25	20	35	23	37	19	41	31	22	16	20

Table S5. KEGG enrichment analysis of differential expression genes in different cross combinations.

Pathway	Pathway ID	Q value															
		AE	AF	AG	AH	BE	BF	BG	BH	CE	CF	CG	CH	DE	DF	DG	DH
Ascorbate and aldarate metabolism	ko00053	/	0.02	/	/	/	/	/	/	/	/	/	/	/	/	/	/
Ubiquinone and other terpenoid-quinone biosynthesis	ko00130	/	/	/	0.02	/	/	/	/	/	/	/	/	/	/	/	/
Photosynthesis	ko00195	0.01	/	/	0.02	0.00	0.00	0.01	0.00	0.00	/	/	0.00	0.00	/	/	/
Photosynthesis - antenna proteins	ko00196	0.00	0.00	0.00	0.00	0.00	0.00	0.00	0.00	0.00	0.00	0.01	0.00	0.03	/	/	0.00
Glycine, serine and threonine metabolism	ko00260	0.01	0.05	0.00	/	/	/	/	/	/	0.02	/	/	0.00	/	/	/
Monobactam biosynthesis	ko00261	0.02	0.01	/	0.02	/	/	/	/	0.02	0.00	/	/	/	/	/	/
Cysteine and methionine metabolism	ko00270	/	0.02	/	/	/	/	/	/	/	/	/	/	/	/	/	/
Lysine biosynthesis	ko00300	/	/	/	/	/	/	/	/	/	/	/	0.04	/	/	0.02	
Arginine and proline metabolism	ko00330	/	/	0.02	/	/	0.05	/	/	0.02	/	/	/	/	/	/	/
Histidine metabolism	ko00340	0.01	/	/	/	/	/	/	/	/	/	/	/	/	/	/	/
Phenylalanine metabolism	ko00360	0.01	0.00	0.00	0.00	0.01	0.04	0.04	0.00	0.02	0.01	/	/	0.00	0.02	/	/
Tryptophan metabolism	ko00380	0.01	0.00	0.03	0.02	/	/	0.04	0.01	/	0.03	/	/	/	0.03	/	/
Glutathione metabolism	ko00480	/	0.02	/	/	/	0.04	/	/	/	0.01	/	/	/	/	/	/
Starch and sucrose metabolism	ko00500	0.03	/	/	/	/	/	/	/	/	/	/	/	/	/	/	/
Amino sugar and nucleotide sugar metabolism	ko00520	0.02	0.01	/	0.04	/	/	0.04	/	/	/	/	/	/	/	/	/
Glycerolipid metabolism	ko00561	0.03	/	/	/	/	0.04	/	/	/	/	/	/	/	/	/	/
Inositol phosphate metabolism	ko00562	/	/	/	/	/	/	/	/	0.03	/	/	/	/	/	/	/
Linoleic acid metabolism	ko00591	0.01	/	/	/	/	/	/	/		/	/	/	/	/	/	/
alpha-Linolenic acid metabolism	ko00592	0.03	/	/	/	/	/	/	/		/	/	/	/	/	/	/

Pathway	Pathway ID	Q value															
		AE	AF	AG	AH	BE	BF	BG	BH	CE	CF	CG	CH	DE	DF	DG	DH
Pyruvate metabolism	ko00620	/	/	/	/	/	/	/	/	0.03	/	/	0.02	/	/	0.03	
Glyoxylate and dicarboxylate metabolism	ko00630	/	/	/	/	/	/	/	/	/	/	/	/	0.01	/	/	/
Carbon fixation in photosynthetic organisms	ko00710	/	0.00	0.00	/	0.00	0.00	/	0.04	0.00	/	/	0.00	0.00	/	/	/
Thiamine metabolism	ko00730	/	/	/	/	/	/	/	/	0.03	/	/	/	/	/	/	/
Vitamin B6 metabolism	ko00750	/	/	0.02	/	/	/	/	/	/	/	/	/	/	/	/	/
Porphyrin and chlorophyll metabolism	ko00860	/	/	/	/	/	/	/	0.02	/	/	/	/	/	/	/	/
Limonene and pinene degradation	ko00903	0.00	0.01	0.00	0.03	/	/	0.01	0.01	0.02	0.04			0.01	0.03		
Carotenoid biosynthesis	ko00906	/	/	/	0.03	/	/	0.04	/	/	/	/	/	/	/	/	/
Nitrogen metabolism	ko00910	/	0.02	/	0.03	/	/	/	/	/	/	/	/	/	/	/	/
Sulfur metabolism	ko00920	0.04	0.02	/	0.02	/	/	/	0.00	0.00	0.03	0.01	0.00	/	/	/	0.01
Phenylpropanoid biosynthesis	ko00940	/	/	0.01		/	/	/	/	/	/	/	/	0.03	/	0.04	/
Flavonoid biosynthesis	ko00941	/	/	0.02	0.03	/	/	/	0.04	/	/	/	/	/	0.02	/	0.02
Stilbenoid, diarylheptanoid and gingerol biosynthesis	ko00945	0.00	0.00	0.00	0.02	0.01		0.00	0.00	0.05	0.03		0.04	0.02	0.01		0.01
Isoquinoline alkaloid biosynthesis	ko00950	/	/	/	/	/	0.04	/	/	/	/	/	/	/	/	/	/
Tropane, piperidine and pyridine alkaloid biosynthesis	ko00960	/	/	0.03	/	/	/	/	/	/	/	/	/	/	/	/	/
Glucosinolate biosynthesis	ko00966	0.01	/	0.00	0.01	/	/	/	0.01	/	/	/	/	/	/	/	/
Metabolic pathways	ko01100	0.00	0.00	0.00	0.00	0.00	0.04	0.01	0.00	0.00	0.03		0.00	0.00	0.01		0.00
Biosynthesis of secondary metabolites	ko01110	0.00	0.00	0.00	0.00	0.00	0.00	0.00	0.00	0.00	0.00		0.00	0.00	0.01	0.00	0.00
Microbial metabolism in diverse environments	ko01120	0.00	0.00	0.00	0.00	0.00	0.00	0.01	0.00	0.00	0.00	0.04	0.00	0.00	0.01	0.00	0.00

Pathway	Pathway ID	Q value															
		AE	AF	AG	AH	BE	BF	BG	BH	CE	CF	CG	CH	DE	DF	DG	DH
Biosynthesis of antibiotics	ko01130	/	/	0.00	/	/	/	/	/	/	0.01	/	/	0.02	/	0.03	0.01
Carbon metabolism	ko01200	0.01	/	0.00	/	0.00	/	/	/	0.00	/	/	/	0.00	/	0.05	0.00
2-Oxocarboxylic acid metabolism	ko01210	/	/	0.02	/	/	/	/	/	/	/	/	/	/	/	0.04	/
Degradation of aromatic compounds	ko01220	/	/	/	/	/	/	/	0.04	/	/	/	/	/	/	/	/
Biosynthesis of amino acids	ko01230	0.01	0.02	0.00	/	/	/	0.04	0.01	0.03	0.02	/	0.05	0.01	/	/	0.00
Ribosome	ko03010	/	/	/	/	/	/	/	/	/	/	0.00	/	/	/	0.00	/
Phosphatidylinositol signaling system	ko04070	/	/	/	/	/	/	/	/	0.05	/	/	/	/	/	/	/
Plant hormone signal transduction	ko04075	/	0.00	0.02	0.03	/	/	/	/	/	/	/	/	/	/	/	/
Plant-pathogen interaction	ko04626	0.00	0.00	0.00	0.03	0.01	0.04	0.01	/	/	0.00	/	/	/	0.02	/	/
Circadian rhythm - plant	ko04712	0.00	0.00	0.00	0.00	0.00	0.00	0.02	0.00	0.00	0.01	/	0.03	/	/	/	0.01

/: Q value > 0.05.

Table S6. KEGG Enrichment analysis of module genes of Chinese cabbage.

Modules	Pathway	Pathway ID	Q values
MM.mediumpurple2	Ribosome biogenesis in eukaryotes	ko03008	0.00
MM.orangered3	Ribosome	ko03010	0.00
	Ribosome biogenesis in eukaryotes	ko03008	0.01
MM.honeydew1	Plant-pathogen interaction	ko04626	0.00
	Glucosinolate biosynthesis	ko00966	0.02

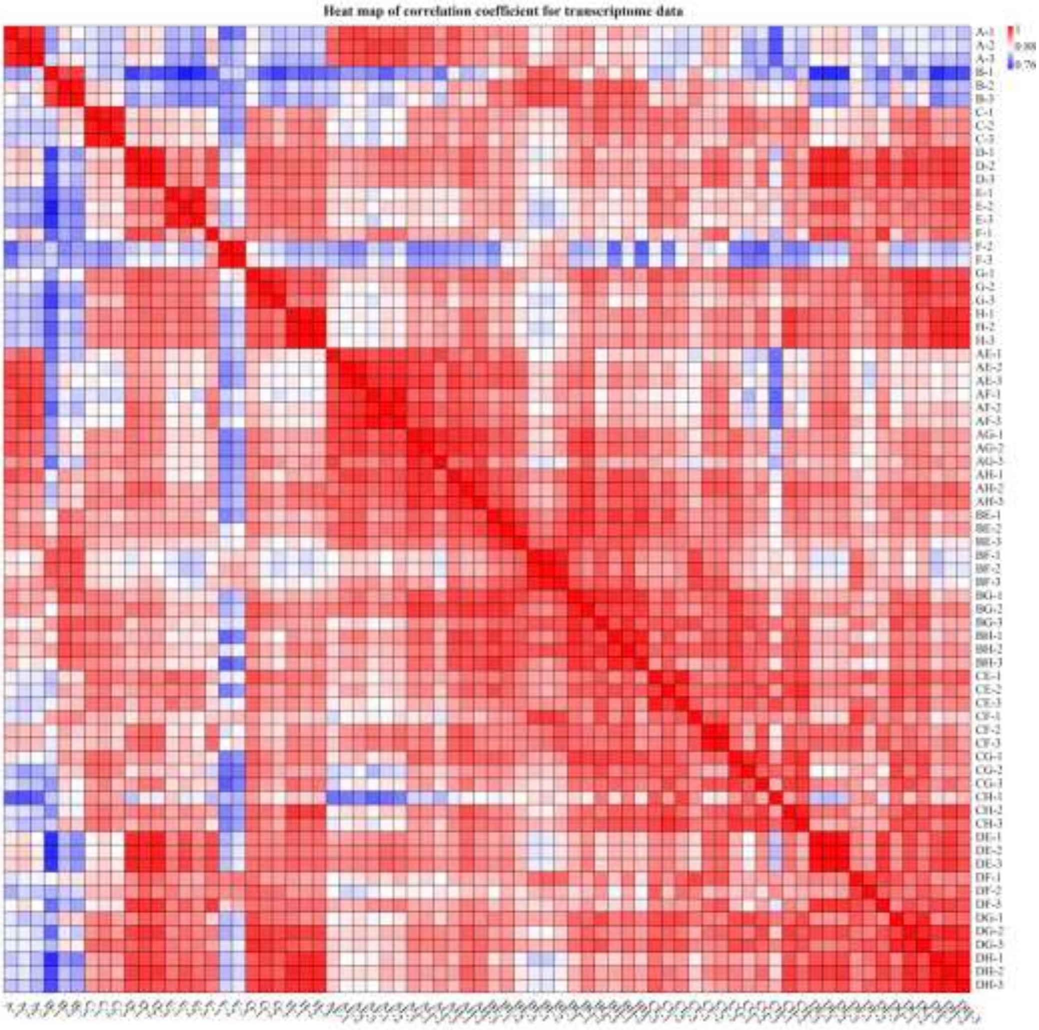

Figure S1. Heat map of correlation analysis of transcriptome data in different samples.
